# Autism Rating Scale: A New Tool for Characterizing the Schizophrenia Phenotype

**DOI:** 10.3389/fpsyt.2021.622359

**Published:** 2021-01-26

**Authors:** Davide Palumbo, Giovanni Stanghellini, Armida Mucci, Massimo Ballerini, Giulia Maria Giordano, Paul H. Lysaker, Silvana Galderisi

**Affiliations:** ^1^Department of Psychiatry, University of Campania “Luigi Vanvitelli”, Naples, Italy; ^2^Department of Psychological, Humanistic and Territorial Sciences, G. D'Annunzio University, Chieti, Italy; ^3^D. Portales University, Santiago, Chile; ^4^Department of Mental Health, Florence, Italy; ^5^Richard L Roudebush Veterans Affairs Medical Center, Indianapolis, IN, United States

**Keywords:** social dysfunction, schizophrenic autism, schizophrenia, remitted schizophrenia, autistic traits, euthymic bipolar disorder

## Abstract

Social dysfunctions (SD) are frequently observed in subjects with schizophrenia. Some of these dysfunctions are also observed in other neuropsychiatric disorders such as autism spectrum disorders (ASD), major depression, bipolar disorder, or Alzheimer disease. Recently, a characterization of a specific type of SD in schizophrenia has been proposed, with the concept of dis-sociality, which form the core aspect of “Schizophrenic Autism” (SA). The present study aimed to explore the presence in people with schizophrenia of SA, independent of other autistic traits, which can be often found in schizophrenia and other neurodevelopmental disorders. We used a structured interview—the Autism Rating Scale (ARS), an instrument devised to detect and measure SA. Fifty-one outpatients affected by schizophrenia (26 remitted, SCZ-r) and 28 affected by bipolar disorder type 1, with psychotic features, in the euthymic phase (BD-e) were recruited. Before assessing the specificity for schizophrenia of SA, we tested the internal consistency, the convergent and divergent validity of the ARS in the schizophrenia sample. Specificity was assessed by examining potential differences in ARS scores between SCZ-r and BD-e subjects. ARS showed good internal consistency, as well as convergent and divergent validity. ARS items were more frequently of moderate severity in SCZ-r than in BD-e subjects. This scale can contribute to establish more precise phenomenal boundaries between schizophrenia and bipolar disorder, and opens up the possibility of identifying a different type of SD in schizophrenia, independent of autistic traits and negative symptoms, which might benefit from different treatments.

## Introduction

DSM-5 (1) defines Social Dysfunction (SD)—within the diagnostic criteria for schizophrenia—as an impairment of social functioning (e.g., interpersonal relationships) and, when the onset of the disorder occurs in adolescence, as the impossibility to reach the expected levels of interpersonal functioning. This conceptualization of SD has three main limitations: (1) it endorses a strictly behavioral-functionalist perspective in which deficits in social behavior are emphasized; (2) these deficits are mainly defined and assessed in terms of quantitative reduction in performance; and (3) the current concept encompasses the real-life functioning domain of impairment, i.e., reduction of social contacts, which might be the consequence of stigmatization (2, 3). Due to these limitations, it is difficult to differentiate SD as a specific dimension of schizophrenia psychopathology from SD in general, or SD that merely emerges in the face of adversities. Several studies on SD in schizophrenia reflect these limitations, as they do not investigate the personal level of experience in the affected subjects. There is a need, therefore, to develop tools assessing the experiential dimension of SD in people with schizophrenia.

### Phenomenological Perspective on Autism in Schizophrenia

Lately, a phenomenological characterization of Social Dysfunction (SD) in schizophrenia has been introduced, with the concept of dis-sociality (4–6). The concept emphasized the subjective alteration of social competence by going beyond the behavioral-functionalist perspective. It reflects a disturbance of participation in social life related to phenomena defining the “Schizophrenic Autism” (SA).

The concept of SA refers to a detachment from reality associated with a rich fantasy life (7) and includes several symptoms and signs, such as emotional indifference, rigid attitude and behavior, dereistic, and overinclusive thinking. The number and variety of included features illustrates the difficulty in defining autism, and reflects the fact that none of these features is in itself sufficient to “diagnose” SA (7, 8). Recently, clinical phenomenologists resumed the construct of SA building on and extending the conceptualizations of Minkowski and Blankenburg. Minkowski et al. (9) assumed that autism is the primary, fundamental disorder in schizophrenia, i.e., a trait alteration from which other psychopathological features of the syndrome originate. He defined autism as the loss of vital contact with reality, an impairment in the capacity to adjust and modify one's own behavior in a contextually relevant manner. Blankenburg (10) characterized autism as a crisis of “common sense”, i.e., the lack of the ability to comprehend “the rules of the game” of human behavior (e.g., the background of tacit knowledge shared by a social group). Common sense is not intended as a body of objective knowledge, but as a natural attitude that underlies the ability to be attuned to the world as it appears in everyday experience. From this perspective, the fundamental anomaly is in the pre-conceptual and pre-cognitive appraisal of social situations (11).

The essential feature of the SA is a qualitative impairment of spontaneous and intuitive participation in social life, referred to as dis-sociality (12). Dis-sociality embraces negative (disturbances of social attunement, detachment form social standards, social shared knowledge, and principles of causality) and positive features (a peculiar set of values), both contributing to the impairment of patients' social attitude (13).

In recent years, research has investigated the relationships between autism spectrum disorders (ASD) and other psychiatric conditions, such as schizophrenia and bipolar disorder (14, 15). In a recent meta-analysis (15), Lai et al. note that schizophrenia and bipolar disorder co-occur in 4% and 5% of cases of ASD, respectively. Several studies have investigated sub-threshold ASD in bipolar disorder and schizophrenia (16–19). Dell'Osso et al. (19) note that ~43% of the sample of subjects with bipolar disorder have clinically significant autistic traits. Studies carried out on samples of patients with schizophrenia, using the PANSS Autism Severity Score (PAUSS), report that a large portion of patients with schizophrenia (40–50%) has clinically significant ASD (16, 17). These data are in line with the hypothesis that autism, schizophrenia, and bipolar disorder may have a common etiology, although they show different psychopathological phenomena (18, 20, 21).

Unlike the ASD traits that can co-occur in different psychiatric diseases, SA is believed to identify characteristic symptoms of schizophrenia. The assessment of ASD and SA is also different: while ASD rating scales mainly evaluate behavior through observation, SA investigation considers exclusively the subjective experience of social life.

### Study Aims

The present study aimed to explore the prevalence of SA in people with schizophrenia and in those with bipolar disorder-type I with psychotic features in a euthymic phase, and to demonstrate its specificity for schizophrenia. We used a structured interview—the Autism Rating Scale (ARS) (11, 22), specifically developed to measure SA. The focus is on persons' experience of social interaction, i.e., their own description about emotional attunement/disattunement, self-other demarcation/non-demarcation, emotion recognition/non-recognition, emotional/cognitive attitude toward others, endorsement/refusal of social norms. A secondary aim of the study was to assess the independence of SA from ASD traits, as measured by the PAUSS, in subjects with schizophrenia.

## Methods

### Study Participants

Fifty-one outpatients affected by schizophrenia (SCZ), and 28 euthymic outpatients with bipolar disorder-type I with psychotic features (BD-e) who experienced one or more recent episodes of depression or mania with psychotic features were recruited from those regularly attending the outpatient unit for psychotic or mood disorders of the Department of Psychiatry of the University of Campania “Luigi Vanvitelli” and consecutively seen from January 2016 to May 2017, who accepted to participate in the study. Inclusion criteria were: (a) diagnosis of SCZ or BD-Type I, according to DSM-IV criteria, confirmed by the Structured Clinical Interview for DSM-IV - Patient Version (SCID - IP); (b) sufficient motivation, introspective skills, and appropriate language skills to participate in the interview, evaluated on the referring psychiatrist clinical impression. Exclusion criteria were: (a) neurological diseases; (b) history of alcoholism or substance abuse; (d) intellectual disability; (e) changes in antipsychotic medication or hospitalization within 3 months prior to the inclusion in the study. For bipolar patients, euthymia was defined as remission of the mood episode and psychotic symptoms for at least 4 weeks at the time of the evaluation.

The study was approved by the Ethics Committee of the University of Campania Hospital and all patients signed an informed consent before being included in the study.

### Instruments

All study participants were assessed by the following instruments:

1. The Italian version of the ARS to assess SA (22). The scale explores the subjective experience of inter-personal relationships, contacts and social situations of people with schizophrenia in their daily life in the last 3 months. It investigates all kinds of real-life situations (e.g., home, work, school, leisure, friendship, etc.), including behavioral aspects (e.g., diminished social interests, interactions, reduced interpersonal involvement, etc.). The ARS includes 16 distinctive items grouped in 6 dimensions: Hypo-Attunement, Invasiveness, Emotional flooding, Algorithmic conception of sociality, Antithetical attitude toward sociality and Idionomia [further information on dimensions in (11)]. Severity is scored on a scale from 1 to 7 (higher scores correspond to greater severity) by taking into account frequency, intensity of subjective arousal or distress, level of impairment, and possibility to cope. The interview takes 30–60 min. In Table 1 the 6 ARS dimensions are shortly described.
2. The Positive And Negative Syndrome Scale (PANSS) is a 30-item clinical scale which evaluates general psychopathology, positive and negative symptoms (23). Each item is rated on a 7-point symptom severity scale, ranging from 1 (absent) to 7 (extremely severe). In this study, ratings on PANSS items were summed to calculate positive and disorganization dimensions of schizophrenia symptomatology, according to the consensus factor model by Wallwork et al. (24).
3. The PANSS autism severity score (PAUSS) is a scale composed by 8 PANSS items, covering the three main specific autism symptom clusters, summed up as follows: (a) difficulties in social interaction: item 1 (“blunted affect”), 3 (“poor rapport”), and 4 (“social withdrawal”) of the PANSS negative subscale; (b) difficulties in communication: items 5 (“difficulties in abstract thinking”) and 6 (“lack of spontaneity and flow of conversation”) of the PANSS negative subscale; (c) limited, repetitive and stereotypic patterns of behavior: items 5 (“mannerism”) and 15 (“preoccupation”) of the PANSS general subscale and item 7 of the PANSS negative subscale (“stereotyped thinking”) (16). Each PAUSS item, according to PANSS, is rated on a 7-point scale and a total score is derived by summing all 8 items (range: 8–56) with higher scores indicating more severe autistic features.
4. The Brief Negative Symptom Scale (BNSS) was administered to evaluate the severity of the negative symptoms: it consists of 13 items organized in 6 sub-scales: anhedonia, distress, asociality, avolition, blunted affect, and alogia. All the items are rated on a 7-point scale (0–6), with a total scores ranging from 0 to 78. For all items in the 6 domains, the highest score is associated with the greatest severity of symptoms, while for the distress item the highest score is associated with the greatest reduction or absence of negative emotions. The total score of the BNSS is calculated by summing the ratings from all the items except for the item “distress”; the scores of the subscales are calculated by summing the scores of the items that the subscale includes. The Italian version of the scale was validated as part of the Italian Network for Research on Psychoses activities (25).

**Table 1:** List and description of the ARS domains.

**ARS domains**	**Items (N)**	**Description**
Hypo-Attunement	3	The immediate feeling of reduced attunement, i.e., emotional contact with other persons. The pervasive feeling of inexplicability /incomprehensibility of people's behavior and social situations.
Invasiveness	3	Feeling oppressed and invaded by the others, from without.
Emotional flooding	2	Feeling oppressed and submerged from within by paroxysms of one's emotions and bodily sensations evoked by interpersonal contacts.
Algorithmic conception of sociality	3	The conceptual, analytic, hyper-cognitive, hyper-rationalist, hyper-reflective stance toward sociality. Patients may endorse a mechanistic, strategic and in some way “mathematisable” (as in a chess game) conceptualization of interpersonal transactions in everyday life.
Antithetical attitude toward sociality	3	Feeling to be vulnerable to the influx coming from the external world and claim one's independence as the most important value.
Idionomia	2	Idionomia is characterized by an existential re-orientation driven by the exaltation of one's own principles, interrogations, or world-view. This exalted existential standpoint does not allow integration or compromise with the other's point of view or with common sense.

### Training of Evaluators and Assessment of Inter-rater Reliability

The assessment was conducted by three residents in Psychiatry properly trained for the administration of the instruments. Both for the PANSS and BNSS the three evaluators achieved a certificated training. The training for the administration of the ARS was conducted by one of the authors of the instrument (MB) and an excellent agreement was observed among raters (intraclass correlation coefficient ranging from 0.74 and 0.96). Further information on the procedure of the training and inter-rater reliability analysis can be found in Ballerini et al. (22).

### Statistical Analysis

All statistical analyses described below were conducted using IBM SPSS Statistics Version 22. The significance level for all statistical comparisons was set at *p* < 0.05.

Before assessing the specificity for schizophrenia of the observed SA and the degree of its association with ASD traits as assessed by the PAUSS in subjects with schizophrenia, we tested the internal consistency and the convergent validity of the ARS.

#### Internal Consistency

The ARS internal consistency was evaluated using Cronbach's Alpha in the SCZ sample.

#### Convergent Validity

In the SCZ sample, ARS convergent validity was assessed by examining its correlations (both total and dimension scores) with the PANSS positive and disorganization dimensions, as well as with the BNSS total score and dimensions. A Bonferroni correction for multiple comparisons was applied to control for type 1 error.

#### Divergent Validity

In the SCZ sample, ARS divergent validity was assessed by examining its correlations (both total and dimension scores) with the PAUSS total and item scores. A Bonferroni correction for multiple comparisons was applied to control for type 1 error.

#### Specificity

The specificity was analyzed by comparing the frequency and severity of the ARS dimensions between SCZ with remitted (r) positive symptoms (SCZ-r) [according to the severity criteria proposed by (26)] and BD-e. The choice of identifying SCZ-r patients and comparing them exclusively to BD-e is due to the need to minimize the clinical differences between the two groups of subjects (i.e., mood symptoms and positive psychotic symptoms). This allows comparing the frequency and severity of ARS dimensions between the two groups of subjects that do not differ with respect to other clinical characteristics. A one-way analysis of variance (ANOVA) was used to test differences between SCZ-r and BD-e with respect to age, education and duration of illness. The two clinical populations were also compared for sex distribution by the χ2 test.

In order to assess differences in the frequency of symptoms, the number of symptoms of at least mild severity (i.e., with a score ≥3) was computed in both groups. Subsequently, the data obtained were compared by the χ2 test.

Differences in symptom severity between the two patient groups were tested using a multivariate analysis of variance (MANOVA), with dimensions of the ARS (Hypo-Attunement, Invasiveness, Emotional flooding, Algorithmic conception of sociality, Antithetical attitude toward sociality and Idionomia) as within-subject factors, and diagnosis as between subject factor (SCZ-r and BD-e). Follow-up univariate ANOVAs for investigation of simple effects were carried out only when significant group main effects or interactions were found in the MANOVA.

## Results

### Socio-Demographic and Clinical Characteristics

The SCZ group was composed by 51 subjects, 33 (64.7%) males, with a mean age of 40.33 (SD ± 10.82), mean education of 13,57 (SD ± 3.05) years and mean illness duration of 17.8 (SD ± 9.96) years.

Twenty-six out of 51 SCZ met the remission criteria. No statistically significant difference was found between the SCZ-r group and BD group for gender distribution (χ^2^ = 0.28; *p* = 0.60), age (*F* = 2.04; *p* = 0.16), education (*F* = 0.67; *p* = 0.41), and duration of illness (*F* = 0.56; *p* = 0.46). Medication, socio-demographic, and clinical characteristics of the study groups are illustrated in Table 2.

**Table 2:** Characteristics of the study groups.

	**SCZ *n* = 51**	**SCZ-r *n* = 26**	**BD-e *n* = 28**
Males (%)	64.7	50	57.14
Age (mean yrs ± SD)	40.33 ± 10.82	37.19 ± 11.63	41.29 ± 9.39
Education (yrs mean ± SD)	13.57 ± 3.05	14.19 ± 3.02	13.25 ± 5.07
Illness Duration (yrs mean ± SD)	17.8 ± 9.96	14.15 ± 9.99	16.29 ± 10.9
**Antipsychotic therapy**			
Typical antipsychotics % (N/Total)	17.65 (9/51)	19.23 (5/26)	10.71 (3/28)
Atypical antipsychotics % (N/Total)	64.71 (33/51)	73.07 (19/26)	85.71 (24/28)
Typical and Atypical antipsychotics % (N/Total)	17.65 (9/51)	7.69 (2/26)	0 (0/28)
No antipsychotic treatment	0 (0/51)	0 (0/51)	3.57 (1/28)
Chlorpromazine – equivalent daily dose – median (Range)	400 mg (125–1,200)	400 mg (125–1,200)	300 mg (0–800)

*SCZ, Subjects affected by schizophrenia; SCZ-r, Subjects affected by schizophrenia with remitted positive symptoms; BD-e, Subjects affected by bipolar disorder-type I with psychotic features during a euthymic phase*.

### Internal Consistency

The internal consistency of the ARS was very high (α = 0.850) suggesting excellent psychometric properties.

### Convergent Validity

The ARS total score was significantly correlated with the positive dimension of the PANSS (*r* = 0.50, *p* < 0.01). The ARS dimensions *Invasiveness, Algorithmic conception of sociality*, and *Idionomia* were moderately correlated with the PANSS Positive dimension (Table 3). The ARS total score had no correlation with negative symptoms (*r* = −0.040, *p* > 0.2), assessed by BNSS; the ARS dimension *Antithetic attitude toward sociality* had a moderate positive correlation with the BNSS total score, due to the correlation with the subscales *Asociality* and *Avolition*, while the ARS dimension *Invasiveness* had a moderate negative correlation with the BNSS total score, due to the negative correlation with the two subscales *Alogia* and *Blunted affect* (Table 3).

**Table 3:** Correlations of Autism Rating Scale total and dimensions scores with other psychopathological dimensions.

**Other Psychopathological dimensions**	**Autism Rating Scale**						
	**Hypo-attunement**	**Invasiveness**	**Cenesthopatic/emotional flooding**	**Algorithmic conception of sociality**	**Antithetical attitude toward sociality**	**Idionomia**	**ARS total**
BNSS anhedonia	0.136	−0.287	−0.070	0.096	0.369	−0.120	0.051
BNSS distress	0.004	−0.234	−0.066	−0.013	0.177	−0.196	0.061
BNSS asociality	0.056	−0.183	−0.109	−0.051	0.413^**^	−0.209	0.003
BNSS avolition	0.085	−0.159	−0.073	−0.023	0.425^**^	−0.236	0.032
BNSS blunted affect	−0.068	−0.304^*^	−0.173	−0.050	0.140	−0.243	−0.146
BNSS Alogia	0.095	−0.383^**^	−0.122	0.064	0.139	−0.095	−0.062
BNSS total score	0.058	−0.319^*^	−0.131	0.008	0.330^*^	−0.217	−0.040
PANSS pos	0.200	0.569^**^	0.250	0.349^*^	0.247	0.474^**^	0.504^*^
PANSS dis	−0.052	−0.050	0.021	0.183	−0.132	0.253	0.104

*BNSS, Brief Negative Symptom Scale; PANSS pos, Positive and Negative Syndrome Scale, positive dimension; PANSS dis, Positive and Negative Syndrome Scale, disorganization dimension*.

* *p < 0.05*;

** *p < 0.01*.

### Divergent Validity

The ARS total score had no correlation with autistic features calculated by PAUSS total score (*r* = 0.095, *p* > 0.2); the ARS dimension *Invasiveness* had a moderate inverse correlation with *Blunted affect, Social withdrawal*, and *Lack of spontaneity*, and the dimension *Antithetic attitude toward sociality* had a low correlation with the PANSS items *Social withdrawal* and *Mannerism*. Only the PAUSS item *Preoccupation* showed moderate correlations with several ARS dimension, except *Hypo-attunement* and *Antithetic attitude toward sociality* (Table 4).

**Table 4:** Correlation coefficients between Autism Rating Scale and PANSS autism severity score (PAUSS).

**PAUSS**	**Autism Rating Scale**						
	**Hypo-attunement**	**Invasiveness**	**Cenesthopatic/emotional flooding**	**Algorithmic conception of sociality**	**Antithetic attitude toward sociality**	**Idionomia**	**Total**
Blunted affect	0.039	**−0.332**	−0.164	−0.008	0.267	−0.143	−0.067
Poor rapport	0.136	−0.144	−0.018	0.101	0.123	0.067	0.063
Social withdrawal	0.026	**−0.470**	−0.166	−0.054	**0.288**	−0.193	−0.128
Difficulties in abstract thinking	−0.060	−0.106	0.198	0.050	−0.008	0.032	0.011
Lack of spontaneity	0.118	**−0.343**	−0.155	0.173	0.158	0.077	0.015
Stereotyped thinking	0.150	−0.141	0.028	0.234	0.092	0.268	0.132
Mannerism	0.142	−0.008	0.049	0.094	**0.276**	0.108	0.159
Preoccupation	0.215	**0.314**	**0.411**	**0.335**	0.090	**0.316**	**0.398**
Total	0.133	−0.242	0.018	0.164	0.230	0.088	0.095

*In bold correlations with a significance level of p < 0.05*.

### Specificity

#### Frequency of Symptoms

**Figure 1:**
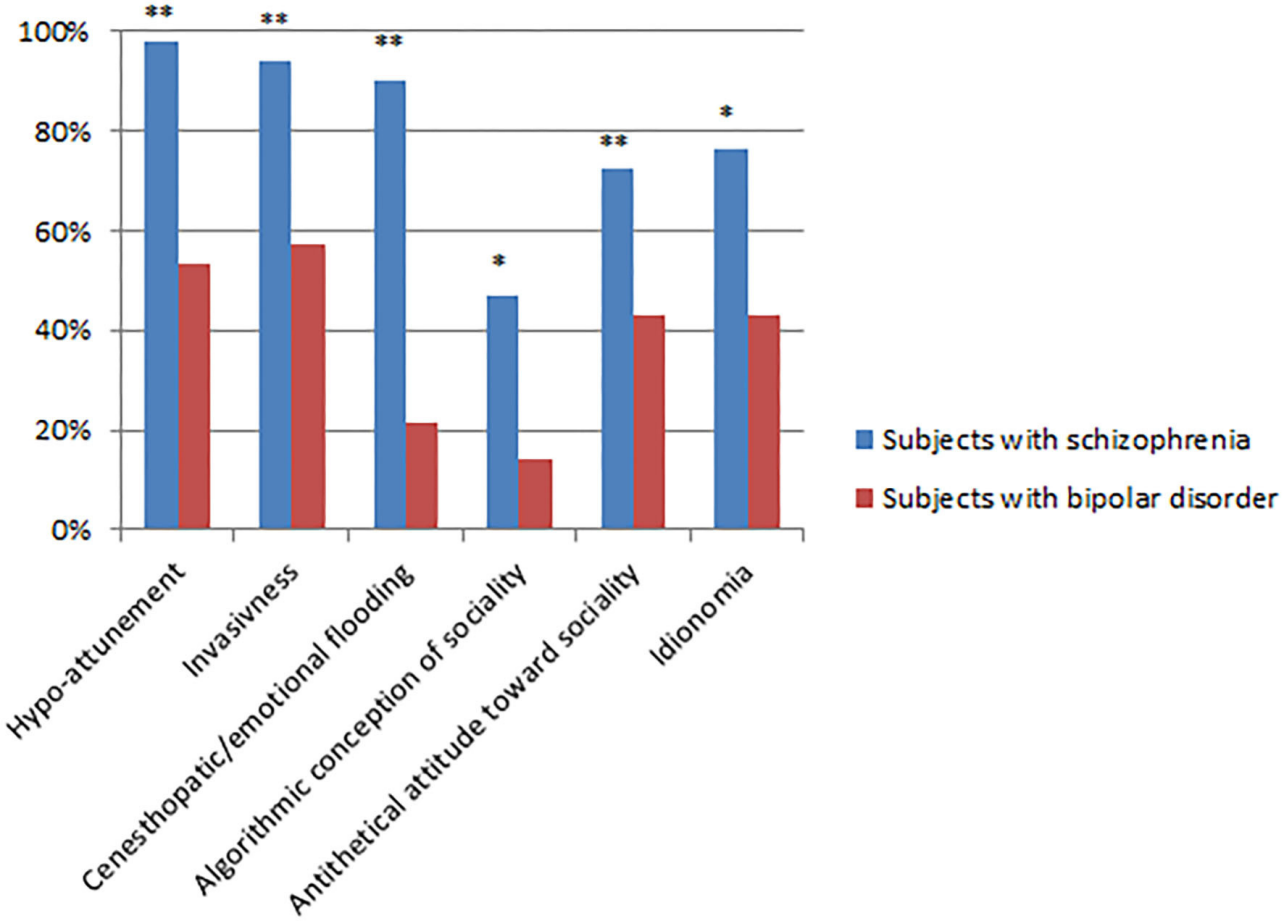
Frequency (%) of ARS dimensions of at least mild severity (≥3); *p < 0.05, **p < 0.01.

The frequency of ARS dimensions showing at least mild severity (i.e., ≥3) (Figure 1) was higher in the SCZ-r than in BD-e sample, except for *Idionomia* dimension (frequency: 65.38% in the SCZ-r sample compared to 42.9% in the BD-e sample, *p* = 0.09). All dimensions had at least mild severity in over 65% of subjects with SCZ-r.

#### Severity of Symptoms

MANOVA showed an interaction group x dimensions (*F* = 10.61, *p* < 0.0001). ARS mean scores were significantly higher in patients with SCZ-r than in those with BD-e on all dimensions (Table 5).

**Table 5:** Severity of the Autism Rating Scale (ARS) scores in the two patient groups.

**ARS**	**SCZ-r (*****N*** **=** **26)**		**BD-e (*****N*** **=** **28)**		***p***
	**Mean ± SD**	**Range**	**Mean ± SD**	**Range**	
Hypo-attunement	11.69 ± 3.31	5–19	4.61 ± 1.66	3–9	0.0001
Invasiveness	10.58 ± 4.74	3–19	5.07 ± 1.98	3–10	0.0001
Cenesthopatic/emotional flooding	7.04 ± 2.58	2–11	3.00 ± 1.51	2–8	0.0001
Algorithmic conception of sociality	7.88 ± 4.79	3–17	3.68 ± 1.02	3–6	0.0001
Antithetical attitude toward sociality	8.38 ± 3.88	3–17	4.18 ± 1.41	3–9	0.0001
Idionomia	5.85 ± 3.56	2–13	3.57 ± 2.00	2–8	0.01
ARS global score	51.42 ± 16.37	25–80	24.11 ± 6.41	17–41	0.000000001

*SCZ-r, Subjects affected by schizophrenia with remitted positive symptoms; BD-e, Subjects affected by bipolar disorder-type I with psychotic features during a euthymic phase; SD, Standard Deviation*.

## Discussion

### Schizophrenic Autism as Assessed by the ARS

The ARS (11, 22) contributed to a detailed characterization of social experiences of SCZ. It documents impairments of the intuitive, pre-reflexive grip on social situations *(Hypo-attunement*), fears of *invasion/violation* of one's own personal space and of being *submerged by own's emotions* when facing other people. Anomalies of intuitive attunement with others may be compensated by the attempt to make sense of the others' behavior and grasp the meaning of social interactions through a hyper-cognitive stance (*Algorithmic conception of sociality*).

Also, the ARS contributes to characterize the patients' social attitude, i.e., their reflexive and deliberate motivation of their asociality and social withdrawal, linking their behavior to a peculiar set of values (12, 27) whose principal features are the refusal of common-sense knowledge and the devaluation of interpersonal bonds (*Antagonomia*), the endorsement of an idealistic quasi-utopian humanitarism *(Abstract idealization*) and the exaltation of idiosyncratic principles and rules, all detached from the values, standards, and symbols characterizing their socio-cultural context (*Idionomia*).

The ARS focuses on these characteristics of SA organizing them in six domains: *Hypo-attunement, Invasiveness, Emotional flooding, Algorithmic conception of sociality, Antithetical conception of sociality*, and *Idionomia*. The internal consistency proved to be excellent (Cronbach' alpha 0.850). These findings demonstrate that the ARS is suitable for clinical assessment and research purposes.

### Convergent Validity

The convergent validity was evaluated in the total sample of SCZ. The ARS total score was correlated with the PANSS positive subscale, and this effect was largely due to *invasiveness, algorithmic conception of sociality* and *idionomia*. All these phenomena contribute to the unusual behaviors occurring in schizophrenia, being connected to the fragility of ego boundaries, a peculiar way to understand others and social situations, and to a radical breakdown of common sense (12, 27).

*Antithetical attitude toward sociality* (*antagonomia and abstract idealization*) was correlated with BNSS “avolition” and “asociality,” suggesting that a peculiar set of values may contribute to negative symptomatology.

The only aspect that was not associated with either positive or negative symptomatology was *Hypo-attunement*. Hypo-attunement refers to a particular impairment of social cognition (SC), distinct from the impairment of the ability to process social information and from the theory of mind (28). Attunement is the pre-reflexive entanglement between a person and a context of worldly significance based on inter-emotionality and inter-corporeality (8). Robust meta-analyses have documented impairment of the other components of SC in people with schizophrenia (29, 30). Relations between SC and negative symptomatology is debated: Sergi et al. (31) described SC as an independent construct, weakly related with negative symptoms; however, more recent studies have reported either the absence of correlation (32, 33), or moderate correlations (34–36). The presence of different components of SC and the use of diverse instruments to assess SC abilities may account for discrepancies of correlations between SC and negative symptoms. *Hypo-attunement*, as measured by the ARS, implements the assessment of the non-strictly “cognitive” component of SC, which seems independent of the negative symptomatology.

### Degree of Overlap and Divergence of SA and ASD

The ARS total score does not correlate significantly with the PAUSS total score. The reason for this is that the two scales are based on different constructs of “autism” and therefore explore different phenomena.

PAUSS is a scale validated in relation to *Autism Diagnostic Observation Schedule*, a semi-structured scale used for diagnostic purposes in ASD (16). PAUSS is therefore able to grasp some aspects related to ASD in different samples of subjects, including SCZ (16, 17).

In contrast, ARS has been developed to capture core characteristics of SA as it is defined in the phenomenological tradition (see *Introduction*) in SCZ. There is a substantial difference between the characteristics of ASD and the concept of SA. The ARS explores the *experiential* dimension of SA aiming to answer the question “What is it like to be with schizophrenia in the social world”? The PAUSS, on the other hand, assesses *behavior* on the basis of *observation* (as for instance is the case for the “interpersonal behavior” items where the interviewer must measure the patient level of “immersion in himself” during the interview), whereas the ARS measures the patients' micro-narratives related to their mental states including their feelings and distressing experiences.

The lack of correlation between the total ARS score and the total PAUSS score therefore indicates that there might be two different profiles of the complex phenomenon called “autism” in SCZ. The PANSS items included in the PAUSS are from the negative symptom subscale and general symptomatology. The ARS does not exclusively capture the aspects linked to the negative symptoms of schizophrenia and in fact correlates significantly with positive symptoms. The results of the correlation between the individual ARS domains and the PAUSS items are not surprising. The *Invasiveness* domain, that is positively correlated with the PANSS positive dimension, showed a negative correlation with the BNSS and PAUSS items investigating negative symptoms. In line with the latter finding, the *Antithetical attitude toward sociality* domain was positively correlated to some items of the BNSS and also slightly correlated to some PAUSS items.

Finally, the correlations between several ARS dimensions and the item “Preoccupation” of the PAUSS are expected. In fact, this PAUSS item investigates patient's interpersonal behavior, in particular the absorption with self-generated experiences, based on what can be observed by the interviewer from an external perspective. ARS investigates interpersonal behavior from the first-person perspective, that is, starting from the patient's subjective experience. It is therefore possible that PANSS “Preoccupation” and different dimensions of the ARS correlate because they investigate similar aspects from different perspectives.

### Specificity

In this study, the specificity of the scale has been assessed matching the SCZ-r patients with the sample of BD patients. This strategy has been adopted a) to remove the possible confounding effect of higher scores on positive symptoms, with the aim to put in evidence possible vulnerability, trait-like characteristics able to differentiate the two clinical populations. Trait-like characteristics (37) are evident “prior to, during, and following periods of clinical symptom exacerbations,” and are thought to reflect the core process of the disease and to be closely related to an “intermediate phenotype” (38).

The ARS mean total score robustly discriminated SCZ-r from BD subjects; if replicated, the phenomenon of SA, as measured by the ARS, might represent a characteristic *pheno-phenotype* or experiential phenotype (39) of schizophrenia and, possibly, of the whole schizophrenia spectrum disorders.

According to these findings, specific trait-like anomalous experiences can discriminate schizophrenia from bipolar disorder.

In our study, not only the ARS total score, but also all its constitutive domains demonstrated diagnostic specificity. SCZ-r obtained higher scores than BD in each ARS domains, with the partial exception of *Idionomia*. The percent of patients who reported a score of at least mild on the ARS items was significantly higher in the SCZ-r sample than in the BD sample. The strongest significance was found for *Hypo-attunement* and *Emotional flooding*, but also *Invasiveness, Algorithmic conception of sociality*, and *Antithetical attitude toward sociality* resulted significant, documenting specific anomalies of intuitive self-other attunement, fears of violation of one's self from outside and of being submerged by own's emotions from within when facing other people, the attempt to grasp the meaning of social interactions through a hyper-cognitive stance and the refusal of common-sense knowledge and of interpersonal bonds. Only the frequency of *Idionomia* did not discriminate SCZ from BD. The result is not surprising: in fact to assess idionomia (22) the interviewer investigates the patients' charismatic orientation (i.e., the certainty to have a special gift or power) with questions like “Did you happen to receive something like a very particular revelation or profound illumination?” or “Did you notice that you have particular characteristics or faculties that other people do not have?” These ideas, which may or may not crystallize in true grandiose delusions, may be present both in schizophrenia and in bipolar disorders (40–42). In our sample, however, the clinical severity of this experience appears to be greater in SCZ-r, although the frequency does not differ significantly.

## Conclusions

Schizophrenia is a complex condition that defies simple description. In addition to psychotic symptoms and the diagnostic criteria identified by the DSMs, the schizophrenia phenotype is also characterized by anomalous subjective experiences that need to be documented and measured through reliable and valid assessment tools.

SA is regarded by phenomenological psychopathology as a hallmark of schizophrenia: patients display a marked tendency toward the constitution of a private world detached from attunement, harmony, and vital contact with social world and the tendency to escape into a private world that is sometimes filled by an efflorescent imaginative inner life, and others haunted by odd and aloof simulacres. The main limit of the phenomenological literature, however, is the lack of valid instruments to collect reliable data. Our findings demonstrate that the ARS represents a valid instrument to capture the experiential phenotype of dis-sociality, distinct from the negative domain of asociality and from other autistic traits. ARS might measure a specific social dysfunction, characterized by anomalies of the pre-reflexive attunement, with profound disorganization of the basic structure of the social life in schizophrenia, which accounts for the bizarreness and detachment from common sense of the affected subjects (27). The scale should now be used in larger sample studies to investigate more specifically whether this type of social dysfunction has different correlates than the autistic traits and the negative domain of asociality. In particular, it should be investigated if ARS indices have any association with deficits of social and non-social cognition, known to be associated with the autistic traits in schizophrenia [e.g., theory of mind impairment and neurocognitive deficits, (43)], as well as with the lack of motivation subtending asociality in schizophrenia (44).

Furthermore, the validity of the scale should be tested longitudinally in subjects characterized by primary negative symptoms (deficit schizophrenia) which do not remit over time and are characterized by a severe impairment of real-life interpersonal relationships (45). The cross-sectional design of our study clearly prevents further inference on this point and represents a limitation.

## Data Availability Statement

The raw data supporting the conclusions of this article will be made available by the authors, without undue reservation.

## Ethics Statement

The studies involving human participants were reviewed and approved by Ethics Committee of the University of Campania Hospital. The patients/participants provided their written informed consent to participate in this study.

## Author Contributions

DP, AM, GS, MB, and SG designed the experiments. DP and AM analyzed the data. DP, GS, and AM wrote the manuscript in consultation with GG, MB, PL, and SG. All authors contributed to the article and approved the submitted version.

## Conflict of Interest

AM has been a consultant and/or advisor to or has received honoraria from Gedeon Richter Bulgaria, Janssen Pharmaceuticals, Lundbeck, Otsuka, Pfizer and Pierre Fabre; she has a patent applied in the field of treatment of schizophrenia. SG has been a consultant and/or advisor to or has received honoraria from Millennium Pharmaceutical, Innova Pharma - Recordati Group, Janssen Pharmaceutica NV, Gedeon Richter-Recordati, Angelini, Lundbeck Italia and Sunovion Pharmarmaceuticals. The remaining authors declare that the research was conducted in the absence of any commercial or financial relationships that could be construed as a potential conflict of interest.
